# Three Cases of Hare Lip: In One of Which the Operation Resulted in Death

**Published:** 1849-03

**Authors:** 


ART. II. *Three Cases of Hare Lip: in one of which the Operation resulted in Death.* Reported by Dr. HAMILTON.

*Case* 1. August 2d, 1848, I operated on the infant son of the Rev. Mr. Wilson, of Elbridge, Onondaga Co., aged five months. The child had a fair constitution, but for a short time had suffered from a mild form of diarrhœa. This continuing slightly after the patient arrived in town, I deferred the operation for a day or two, when, the diarrhœa having sensibly abated, I operated. The fissure was single and extended through the hard and soft palate. I operated by closing the wound with common interrupted sutures, two large and two small, but substituting "collodion" plaster instead of the "patent." At the end of forty-eight hours I removed three of the sutures and the fourth on the fourth day. The wound healed kindly and union was complete by the fifth day. I renewed the plasters after the third, every other day, until the seventh. The weather was during this time, very warm, and the diarrhœa had continued in a still abated form up to the night of the seventh. He had taken food regularly, yet on the night of the seventh day he looked unusually pale and seemed languid; on the morning of the eighth he awoke apparently as well as usual, but on attempting to feed him, it was noticed that he could not swallow. In a very few minutes, and before I could reach the house, he gradually sank away and died.

A *post mortem* was cheerfully granted by the intelligent parents, and with Dr. Flint, I made an examination in the morning of the same day.

*Autopsy.*—Ventricles of brain containing about four ounces of serum, and no other diseased manifestations within the cranium. The viscera of the thorax were sound, and also of the abdomen, save that at seven distinct points of the small intestines an intussusception existed, involving at each separate point from two to twelve inches. The intussusception, we believe, must have occurred in the article of death, since the bowels had continued to move occasionally, once in eight or ten hours, until the evening preceding.

This case is given as one illustration of the fact that no operation can be made with the same safety upon an infant as upon an adult: for, although the season was warm and the child was actually suffering under a mild diarrhœa, yet it was too mild to have in itself proved a cause of death. The intussusception we mention as a singular circumstance, but it did not produce death, nor do we think that the serous effusion in the brain was anything more than one of the consequences of the general irritation and consequent exhaustion. In short this child died in consequence of the irritation of an operation in connection with a mild diarrhœa; and it ought to teach us not to operate generally upon infants when suffering under any derangement of the bowels, nor perhaps during that season of the year when they are especially liable to such derangements. It is one illustration also, as we have above said, that, contrary to the opinion of some good surgeons, there is not the same safety in operating for hare-lip upon an infant as upon an adult; for the same causes which have produced death here would not have been sufficient to produce death in an adult. This truth ought then be fairly given to the parents before an operation is made.

*Case* 2. On the same day I operated upon the infant daughter of Mr.

——— of Seneca Falls—aged three months. This child had also a slight diarrhœa. The cleft was very wide and extended through the hard and soft palate. The bone on the right side of the cleft had thrown off once or twice a slight exfoliation, and the mucous membrane over it was not healthy. I used in the operation the same number of sutures and the same dressings as in the first case. I removed three sutures on the second day and the fourth on the fourth day. The second time I applied the collodion plaster the child strangled, and afterwards I used common adhesive plaster. She bled pretty freely in the operation, much of which blood being swallowed, increased the diarrhœa. About the eighth day an erysipelas attacked the lips, producing an ulceration at the upper part of the wound which soon dissolved the union at that point. The erysipelas rapidly spread over the whole face and scalp, and the child was with difficulty saved.

This case is scarcely less instructive than the first and ought also to impress upon us the greater hazard of early than of late operations: not that erysipelas may not attack a wound made at any period of life, and at least twice this has happened with me in operations for hare-lip made upon adults, but because when it does occur it is much more likely to prove fatal to the infant than to the adult.

This, as my pupils know, is no new doctrine with me, but it is what I have always taught and still firmly believe, although Dr. Mutter and Dr. Mason Warren, whose experience has been considerable, think differently.

I do not however advise waiting longer than the second year: because although there is still more danger than would exist at a later period, yet the danger is so greatly diminished that it is now quite outweighed by the fact that the earlier the operation is made, the more complete will be the restoration of both the lip and palate. *The period immediately preceding or succeeding dentition ought generally to be preferred.*

*Case* 3. Mary ———, aged thirteen months—Double hare-lip. Last winter I operated upon this child. There was then a double fissure, and the central piece of integument was pushed forward by bone so that it lay on the same plane with the ridge of the nose; this piece of integument was too short and too narrow to make use of it in supplying the deficiency in the lip. I removed the bone, and then carried the skin covering it directly back, so as to form of it a columna nasi, and secured it in place with sutures.

Sept. 26, 1848, I operated again. Dissecting up the lip on each side, and abrading the edges, I drew it together with four strong interrupted sutures: over this common adhesive straps were applied without compress or roller. The plasters held so firmly that I did not disturb them, or renew the dressing in seven days. The patient was then, without my knowledge, carried home, some fifteen or twenty miles in the country. The parents thought, as I understand, that the cure was completed and that nothing more was to be done. I have only learned since that the case did well. When or how the sutures came out, I have not been informed.

